# Fatness and Fluctuating Body Weight: Effect on Central Vasculature

**DOI:** 10.1089/biores.2017.0044

**Published:** 2018-06-01

**Authors:** Zachary S. Zeigler, Natasha Birchfield, Karen Moreno, Darith James, Pamela Swan

**Affiliations:** ^1^ College of Science, Engineering, and Technology, Grand Canyon University, Phoenix, Arizona.; ^2^ School of Nutrition and Health Promotion, Arizona State University, Phoenix, Arizona.

**Keywords:** arterial stiffness, central blood pressure, obesity, visceral fat, weight cycling

## Abstract

Weight Cycling (WC) is a prevalent behavior associated with adverse cardiovascular (CV) health. However, a 2010 review on the effects of WC and blood pressure (BP) determined that there was not enough evidence to draw definitive conclusions. Central BP is the principal predictor of CV risk compared to peripheral BP. The influence that WC may have specifically on central BP is unknown. Cross-sectional observation of self-reported history of WC on measures of CV health was undertaken. Seventy-five women completed a Weight and Lifestyle Inventory questionnaire, which is considered a reliable index of WC (*r* = 0.87, *p* < 0.001). Measures of visceral fat, BP, arterial stiffness, and VO_2_peak were taken. Regression equations were used to assess primary predictors of these outcomes. Seventy-five middle aged (39 ± 11 years), obese (32 ± 7 kg/m^2^), and relatively unfit (24 ± 8 ml·kg^−1^ min^−1^) women completed the study. Visceral fat was the strongest predictor of brachial systolic blood pressure (SBP; *r*^2^ = 0.283), brachial diastolic blood pressure (DBP; *r*^2^ = 0.176), central SBP (*r*^2^ = 0.375), and augmentation index (AIx; *r*^2^ = 0.535, all *p* < 0.001). VO_2_peak was the strongest predictor of central DBP (*r*^2^ = 0.062, *p* = 0.036) and augmentation pressure (AP; *r*^2^ = 0.491, *p* < 0.001). Weight cycling index was associated with visceral fat (*r* = 0.521, *p* < 0.001). Visceral fat was a mediator between WC and central SBP (confidence interval [CI] = 0.0053–0.0602), AP (CI = 0.0507–0.4915), AIx (CI = 0.0025–0.0699), and carotid-femoral pulse wave velocity (CI = 0.0115–0.1227; all *p* < 0.05). WC may increase visceral fat accumulation, which was associated with increased central SBP and measures of arterial stiffness.

## Introduction

Approximately one of every three deaths in the United States can be attributed to cardiovascular disease (CVD).^1^ Obesity is often cited as the cause of CVD, while weight loss is being advocated as the remedy.^2^ With ∼36% of adults in the United States considered obese,^3^ dieting is prevalent across the nation. The NHANES 2003–2008 survey found that 57% of woman had been on some type of weight loss diet in the preceding year.^4^ However, recidivism rates of weight loss are extremely high.^5^ Anywhere from 80%^6^ to 95%^7^ of those who lose weight are NOT able to sustain it. Therefore, most dieters relapse into a pattern of weight loss and subsequent weight regain sequences termed as weight cycling (WC). Although there is some controversy surrounding the topic of WC,^8,9^ sustained body of research shows that WC is associated with enhanced weight gain in the future^10–12^ and a redistribution of body fat toward an unfavorable phenotype.^13–15^ WC has also been named as a possible contributor of increased blood pressure (BP).^11,12,14,16,17^ However, a 2010 review on the effects of WC and BP in overweight/obese adults concluded that there was not enough evidence of acceptable quality to draw any definitive conclusions.^18^

Growing evidence from epidemiological^19,20^ and clinical observation^21^ suggests that central BP may be more relevant than peripheral BP in predicting target organ damage and cardiovascular (CV) outcomes. While brachial BP is assumed to represent central BP, central BP is more complex and is affected by arterial stiffness and the timing and magnitude of the pressure wave.^22,23^ In fact, certain pharmacological therapies have shown to affect brachial and central BP/hemodynamic properties differently.^24^ Importantly, central BP is the principal predictor of CV risk compared to peripheral BP.^19–21^ Thus, highlighting the importance of knowing both brachial and central BP. The possible influence that WC may have specifically on central BP is unknown.

Indeed, valuable information is neglected when measuring BP at only the brachial artery with the sphygmomanometer method.^24^ As stated above, aortic systolic pressure is determined by both cardiac factors (stroke volume and ejection time) and arterial factors (arterial stiffness and pulse wave reflection). Arterial stiffness increases as elastic fibers in the lamina media of the aorta are destroyed and replaced by collagen fibers^25^ resulting in an increase in aortic impedance and pulse wave velocity (PWV).^26^ The Framingham Heart Study showed that aortic stiffness, measured as carotid-femoral pulse wave velocity (cfPWV), was superior to brachial arterial stiffness, augmentation index (AIx), central pulse pressure, and pulse pressure amplification.^27^ Carotid femoral PWV is now considered the gold standard for measuring arterial stiffness.^28^ Pulse wave analysis (PWA) can give an AIx, which is an indirect measure of arterial stiffness that assesses pulse wave reflection and calculates how much of the central pressure is accounted for by the reflected wave pulse. This can also be expressed in absolute terms as the augmentation pressure (AP). Although PWV and AIx are correlated, they are two different measurements of the properties of the arterial tree that cannot be used interchangeably.^29^

Wildman et al. showed that weight change was associated with congruent changes in PWV.^30^ In that study, the effect of weight change on arterial stiffness was much stronger than the effect weight change on BP. It is now understood that central arterial stiffness precedes the development of hypertension (HTN).^31,32^ To our knowledge there is no study examining the effects of WC on arterial stiffness. Prior research that has failed to find harmful effects of WC on BP has concluded that WC is a benign activity, when in fact, WC may have deleterious effect on arterial stiffness that has not translated to increased brachial BP.

The purpose of this study was to examine the independent effects of fatness (using visceral fat) and self-reported history of body WC on the central vasculature. It was hypothesized that those with a history of WC will have increased arterial stiffness and visceral fat compared to those reporting fewer WC.

## Materials and Methods

### Subjects

Healthy overweight or obese nonsmoking weight stable (maintained current body weight during the past 4 months) Caucasian women with a body mass index (BMI) >25 kg/m^2^ and aged 25–60 years were recruited for this cross-sectional study. Women were recruited from e-mail distribution lists that were sent to Arizona State University campuses. Woman were excluded if they had a BMI <25 kg/m^2^ or if they answered positively (i.e., yes) on The Physical Activity Readiness Questionnaire (PAR-Q). In addition, those with known CV, pulmonary, renal, peripheral vascular disease, or metabolic disease or having symptoms suggestive of these diseases were excluded from the study. Women who reported taking medications for high BP or other medications that would impact arterial stiffness were also excluded. Pregnant woman, current smokers, or anyone with contraindications to vigorous exercise were excluded from the study as well. All procedures were approved by the Arizona State University Institutional Review Board, and written informed consent was obtained from participants before participation.

### Study design

Subjects were asked to come to the research laboratory at the Arizona State University Downtown campus between the hours of 0600 and 0900 for one visit. Subjects were fasted for at least 8 h, refrained from caffeine and alcohol for 24 h, and were asked to abstain from unaccustomed physical activity (PA) for 48 h before their visit to avoid potential carryover effects from previous PA. Upon arriving at the laboratory, anthropometric measures of body weight, height, waist, and hip circumferences were measured. Height was measured with the subject standing barefoot to the nearest 0.1 cm using a wall mounted stadiometer (Seca, Chino, CA). Body weight (kg) was measured using a digital scale (Tanita, Arlington Heights, IL).

Body composition was assessed by dual-energy X-ray absorptiometry (iDXA; General Electric, Fairfield, CT). For the iDXA assessment, subjects were asked to wear metal-free clothing while lying face up on the iDXA scanner bed for approximately 7–10 min. A certified radiography technician conducted all iDXA scans. All participants were asked to take a urine pregnancy test before testing.

#### PWV/pulse wave analysis

Upon completion of the dual-energy X-ray absorptiometry (iDXA) scan subjects were directed to lie in the supine position in a quiet temperature-controlled room. After 20 min of rest and two sequential brachial BP measurements that confirmed hemodynamic stability, central/brachial BP, cfPWV, and PWA measurements were taken using the SphygmoCor XCEL^™^ (AtCor Medical, Sydney, Australia) by means of validated methodology.^33^ This newer XCEL device has been shown as valid and reliable^34^ and uses a simpler, more convenient approach to measure arterial stiffness and wave reflection characteristics.

PWA was performed by deriving an ascending aortic pressure waveform from the brachial artery waveforms recorded at the arm, using a specialized cuff containing a high-fidelity micromanometer. After 10 sequential, high-quality waveforms (software derived from an algorithm, including average pulse height, pulse height variation, diastolic variation, and the maximum rate of rise of the peripheral waveform) are acquired, a validated generalized transfer function was used to generate the corresponding central aortic pressure waveform. BP cuffs were adjusted appropriately to match arm circumference. The AIx was calculated as the difference between the first and second systolic peaks of the ascending aortic waveform expressed as a percentage of the central pulse pressure (the difference between central systolic and diastolic pressure). Because AIx is influenced by heart rate (HR), an index normalized for a HR of 75 beats/min (AIx@HR75) was used.

Carotid-femoral PWV was determined by simultaneously recording carotid artery wave forms by applanation tonometry and femoral artery waveforms using a specialized micromanometer equipped cuff. Distances from the carotid sampling site to the suprasternal notch and from the suprasternal notch to the femoral cuff were measured as straight lines between the points on the body surface using a tape measure. The distance from the femoral arterial pulse to the femoral cuff was obtained and subtracted from the total distance (*D*). The time (*t*) between the onset of femoral and carotid waveforms was determined as the mean from 10 consecutive cardiac cycles. The cfPWV was calculated from the distance between measurement points and the time delay (*t*) as follows: cfPWV = *D*/*t* (m/sec), where *D* is distance in meters, and *t* is the time interval in seconds.

#### VO_2_peak assessment

All subjects performed a ramp style maximal exercise test on a cycle ergometer. Subjects were equipped with a mask attached to a hose connected to a mixing chamber for the metabolic measurement device (Parvo Truemax 2400TM; Parvomedics, Sandy, UT) to measure ventilation and respiratory gas exchange data and wear a Polar HR monitor to measure HR continuously. The Parvo Truemax 2400TM has shown high validity and reliability.^35^ The machine was calibrated using the standard three-point calibration before each test. After collecting resting data for 2 min, subjects performed a 5-min warm-up of 25 W on the cycle ergometer, pedaling at a cadence of their choice (subjects were encouraged to maintain this cadence for the remainder of the test). After the warm-up phase, load increased by 15 W every minute until the subject could not continue. Verbal encouragement was given to all subjects throughout the entire test. The average of the two highest consecutive 15 sec oxygen uptake averages during the test was taken as the peakVO^2^.

#### Measures of WC

Two main measures of WC were used in this study, the first being The Weight and Lifestyle Inventory (WALI) questionnaire.^36^ The WALI has been shown to be a reliable representation of subjects reporting number of diets (test-retest reliability *r* = 0.77) and total amount of weight lost (test-retest reliability *r* = 0.87, both *p* values <0.001).^37^ The questionnaire asks questions related to subjects' weight history, family weight history, pregnancy history, tobacco (current smokers were excluded from the study) and alcohol use, eating habits/patterns, PA behaviors, and medical history. Subjects were asked to record all diets they had undertaken that resulted in a weight loss of ≥10 lb (4.5 kg); this value was considered a WC. These data allowed each subject to be assigned a number of WCs. Weight loss associated with menstrual cycle, illness, or pregnancy was excluded. Subjects reviewed their responses with one of the authors and explained any uncertainties.

Subjects also responded to a series of questions on weight variability. Subjects completed a grid that asked for the number of pounds lost and gained and frequencies of intervals, for example, 1–5, 6–10 lb, 1–5 times, and so on. A weight cycling index (WCI) category was determined by computing the mean weight loss by the number of times attempted.^13^ Frequency of weight loss within defined intervals was given a rank: never = 1, 1–5 times = 2, 6–10 times = 3, etc. Amount of weight loss was calculated as the median of the range of each category on the grid (3, 8, 13, 18, 30, and 63). By multiplying the scores, a continuous WCI was constructed as median amount of weight lost × rank number of times weight lost to form one composite score for each subject. Weight loss associated with menstrual cycle, illness, or pregnancy was excluded from the calculation of the WCI.

### Statistical analyses

All statistical analyses were performed using SPSS software version 21 (SPSS 21.0; IBM Corporation, Armonk, NY). Data are expressed as mean ± standard deviation unless otherwise specified. Data were analyzed for normality, and values with skewed or kurtotic distributions were transformed to achieve normality. Descriptive statistics was used for the demographics of the participants. All *p* values were calculated assuming two-tailed hypothesis, and *p* < 0.05 was considered statistically significant. Bivariate Pearson and Spearman correlations were used to assess if linear relationships existed between WC measures and outcome measures. Independent variables of age, visceral fat, VO_2_peak, and number of WC or WCI that were found to be significantly related to measures of central arterial stiffness were entered into a stepwise multiple linear regression analysis to assess independent effects of each. Due to the collinearity of BMI and visceral fat and the robust data illustrating the deleterious impacts of visceral fat on health, visceral fat was used in place of BMI as an independent variable. There was one subject with a visceral fat of roughly 5000 cm^3^. Data were analyzed both with and without this data point to ensure that this one subject was not driving the association. The *p*-values were not statistically different when analyzing without this data point so this subject was included in the analysis.

Adjusted *r*^2^ values are presented accounting for sample size. Bootstrapping method with bias-corrected confidence interval (CI) was used to run mediation analysis with visceral fat and VO_2_peak as possible mediators. The 95th CI of the indirect effect was obtained with 5000 bootstrap resamples. “A” path was assessing effects of WC on visceral fat and VO_2_peak, “B” path was assessing effects of visceral fat/VO_2_peak on arterial stiffness, and “C” path was assessing the effects of WC on arterial stiffness (direct effect). Age was used as a covariate in all mediation analysis.

## Results

Table 1 shows subject characteristics. Subjects were 75 middle aged (39 ± 11 years), obese (32 ± 7 kg/m^2^), and relatively unfit (24 ± 8 ml·kg^−1^·min^−1^) women. Table 2 shows unadjusted correlations between measures of WC, visceral fat, fitness, and measures of BP and arterial stiffness.

**Table 1.** **Subject Characteristics**

Body mass index (kg/m^2^)	32 ± 7
Body weight (kg)	87 ± 22
Age (years)	39 ± 11
VO_2_peak (ml·kg^−1^·min^−1^)	24 ± 8
Body fat (%, iDXA)	43 ± 8
Visceral fat (cm^3^, iDXA)	1082 ± 885
Brachial SBP/DBP (mm Hg)	122 ± 13/75 ± 9
Central SBP/DBP (mm Hg)	111 ± 13/79 ± 22
AP (mmHg)	8 ± 6
AIx (%)	16 ± 16
PWV (m/sec)	7 ± 2
Number of WC	3 ± 2

*N* = 75.

AIx, augmentation index; AP, augmentation pressure; DBP, diastolic blood pressure; iDXA, dual-energy X-ray absorptiometry; PWV, pulse wave velocity; SBP, systolic blood pressure; WC, weight cycling.

**Table 2.** **Correlations Between Measures of Weight Cycling, Fatness, and Fitness and Measures of Blood Pressure and Arterial Stiffness**

	Visceral fat	VO_2_peak	Brachial SBP	Brachial DBP	Central SBP	Central DBP	AP	AIx	PWV
Visceral fat	1.000	−0.732^a^	0.400^a^	0.400^a^	0.625^a^	0.473^a^	0.663^a^	0.707^a^	0.580^a^
Age	0.421^a^	−0.507^a^	0.275^b^	0.103	0.390^a^	0.198	0.657^a^	0.579^a^	0.569^a^
VO_2_peak	−0.732^a^	1.000	−0.416^a^	−0.290^a^	−0.539^a^	−0.374^a^	−0.718^a^	−0.712^a^	−0.576^a^
WCI	0.521^a^	−0.386^a^	0.146	0.166	0.249^b^	0.196	0.354^b^	0.315^b^	0.345^b^
WC	0.427^a^	−0.335^b^	0.104	0.069	0.200	0.151	0.319^b^	0.363^b^	0.305^b^

^a^ Correlation is significant at the 0.01 level.

^b^ Correlation is significant at the 0.05 level.

WCI, weight cycling index.

Both measures of WC were significantly associated with visceral fat (WCI; *r* = 0.521, WC; *r* = 0.427, both *p* < 0.001), fitness (WCI; *r* = −0.386, WC; *r* = −0.335, both *p* < 0.05), and measures of arterial stiffness (all *p* < 0.05). WC was not associated with brachial BP (*p* = 0.40).

Independent variables with a significant linear relationship to measurers of arterial stiffness were added to a stepwise linear regression (WCI, number of WC, age, visceral fat, and VO_2_peak). Only the WC measure with the highest *R*-value was used in the regression.

**FIG. 1.**
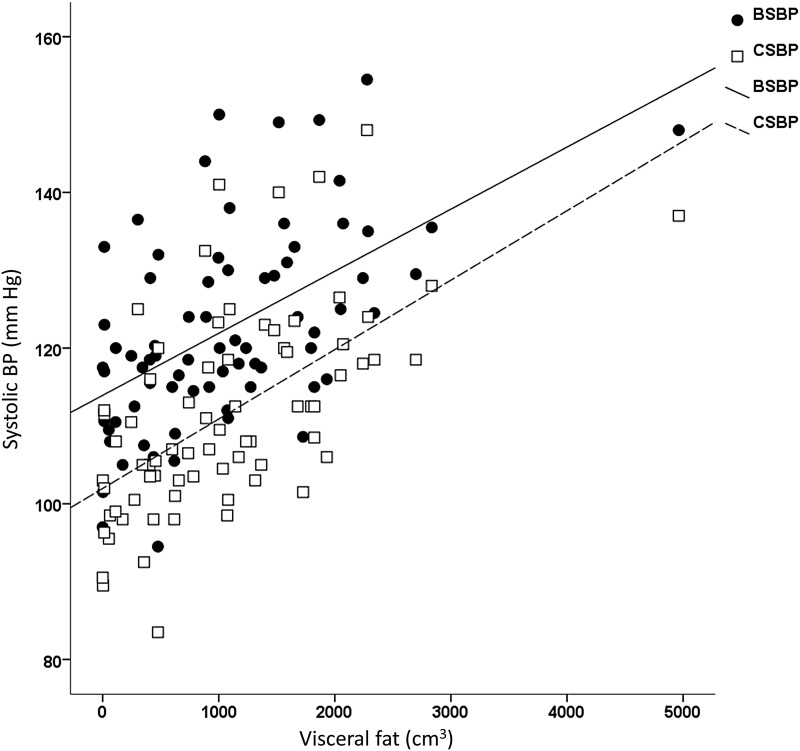
Predictive power of visceral fat (cm^3^) on both peripheral SBP and central SBP (mm Hg). Visceral fat measured using iDXA. *p* < 0.001. SBP, systolic blood pressure.

Figure 1 shows the predictive value of visceral fat on peripheral and central systolic blood pressure (SBP). Visceral fat was the only significant predictor of brachial SBP explaining 28.3% and brachial diastolic blood pressure (DBP) explaining 17.6% (both *p* < 0.001). The strongest predictor of central SBP was visceral fat (*r* = 0.612; *p* < 0.001), with only age significantly adding to the model by explaining 4.8% more of the variance (*p* = 0.024). The only independent predictor of central DBP was VO_2_peak (*r* = −0.249, *p* = 0.036); visceral fat and age did not significantly explain any more of the variance.

**FIG. 2.**
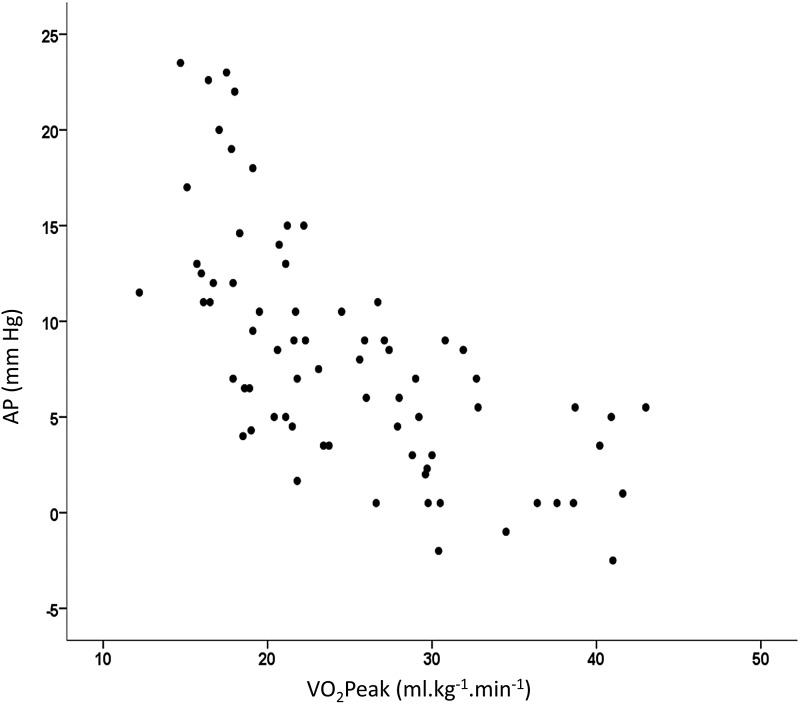
Predictive power of VO_2_peak (ml·kg^−1^·min^−1^) on AP. Correlation significant at *p* < 0.001. AP, augmentation pressure.

VO_2_peak (*r*^2^, 0.491), age (*r*^2^ change, 0.118), and visceral fat (*r*^2^ change, 0.047) all significantly fit the regression equation explaining 64% of the variance of AP (*p* < 0.001). Figure 2 shows the robust association between VO_2_peak and AP with VO_2_peak explaining ∼50% of the variance.

**FIG. 3.**
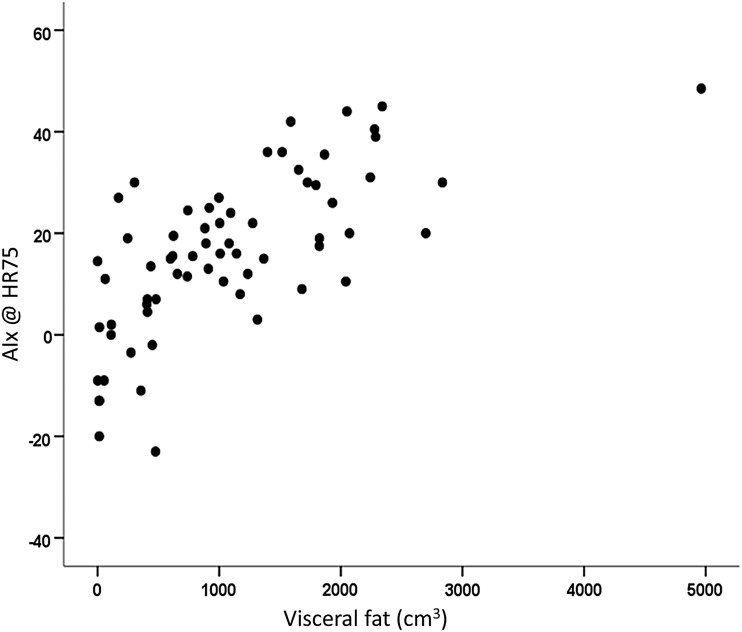
Predictive power of visceral fat (cm^3^) on AIx@HR75. Visceral fat measured using iDXA. *p* < 0.001. AIx, augmentation index; HR, heart rate.

The variables of visceral fat (*r*^2^, 0.535), age (*r*^2^ change, 0.095), and VO_2_peak (*r*^2^ change, 0.024) all significantly fit the regression equation explaining 64% of the variance of AIx@HR75 (*p* < 0.001). Figure 3 illustrates that visceral fat was the strongest predictor explaining ∼54% of the variance.

**FIG. 4.**
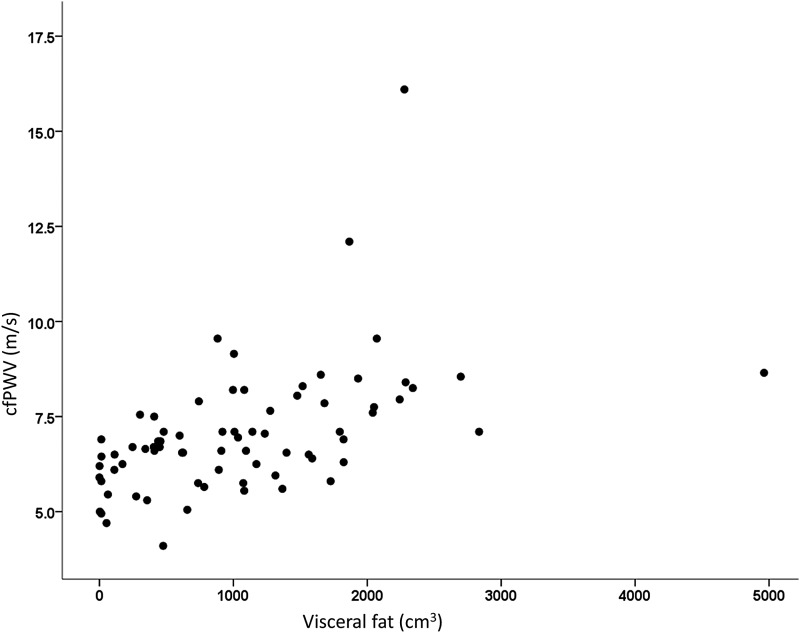
Predictive power of visceral fat (cm^3^) on cfPWV (m/sec). Visceral fat measured using iDXA. *p* < 0.001. cfPWV, carotid-femoral pulse wave velocity.

Only age (*r*^2^ 0.325) and visceral fat (*r*^2^ change 0.124) fit the regression equation explaining 43% of the variance of cfPWV (*p* < 0.05) (Fig. 4).

**FIG. 5.**
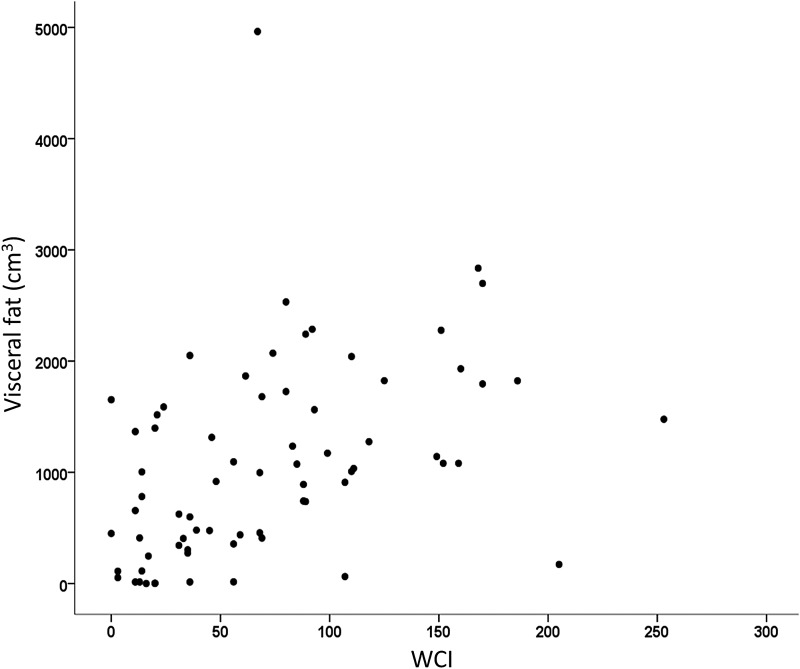
Predictive power of WCI on visceral fat (cm^3^). Visceral fat measured using iDXA. *p* < 0.001. WCI, weight cycling index.

Figure 5 shows the significant association between WCI and visceral fat (*r* = 0.521, *p* < 0.001). VO_2_peak, age, body fat, and WCI were placed into a regression equation to explain variance in visceral fat. After total body fat (*r*^2^, 0.719), only WCI significantly added to the equation (*r*^2^ change, 0.050, *p* = 0.041).

**FIG. 6.**
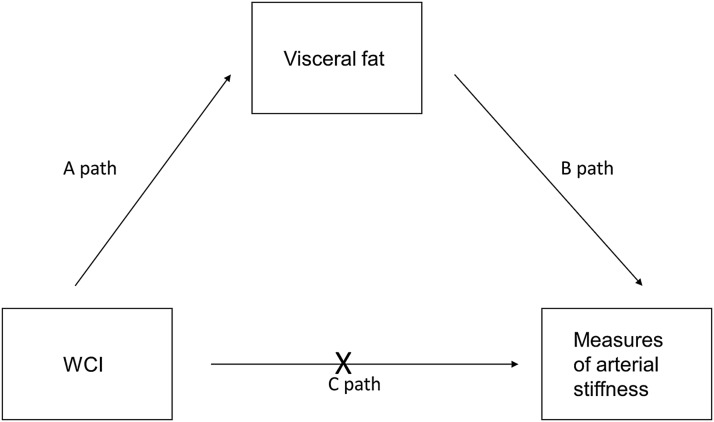
Mediation analysis examining the indirect effects of WC on measures of arterial stiffness (central SBP, AP, AIx@HR75, and cfPWV) through the mediator of visceral fat. C path becomes nonsignificant when adjusting for visceral fat (*p* > 0.05). WC, weight cycling.

Because WC predicted visceral fat and visceral fat predicted measures of arterial stiffness, a mediation analysis was conducted. Figure 6 shows the proposed model to assess if WC exerted indirect effects on arterial stiffness through the mediator visceral fat. Age and fitness were used as covariates. WCI was positively associated with visceral fat [A path; B = 77, *t*(73), *p* = 0.004]. It was also found that visceral fat was positively associated with central SBP [B = 0.0075, *t*(68) = 4.3, *p* < 0.001], AP [B = 0.0035, *t*(73) = 5.8, *p* < 0.001], AIx@HR75 [B = 0.0035, *t*(73) = 5.8, *p* < 0.001], and cfPWV [B = 0.0008, *t*(73) = 3.6, *p* = 0.006; B path]. Because both A path and B path were significant, mediation analysis was tested. Results of the analysis confirmed the mediating role of visceral fat in the relation to WC and central SBP (CI = 0.0053–0.0602), AP (CI = 0.0507–0.4915), AIx@HR75 (CI = 0.0025–0.0699), and cfPWV (CI = 0.0115–0.1227; all *p* < 0.05). In addition, the direct effects of WC on measures of central arterial stiffness (C path) became nonsignificant when controlling for visceral fat, suggesting full mediation (*p* > 0.05).

## Discussion

The 2007 guidelines on the management of arterial HTN state that the relationship between arterial stiffness and negative events is continuous.^38^ In addition, growing evidence suggests that central BP is more relevant than peripheral BP in predicting target organ damage and CV outcomes.^19–21^ Thus, identifying behaviors that negatively affect these measures should be high priority for health professionals.

This is the first study to assess the possible effects of WC on arterial stiffness. The major finding of this investigation was that after adjusting for age and fitness, visceral fat, not WC, was the strongest predictor of increased brachial SBP/DBP, central SBP, and AIx@HR75. After age, visceral fat, not WC, was the strongest predictor of PWV. Higher levels of WC, however, were predictive of increased levels of visceral fat. Thus, visceral fat was found to be a mediator between WC and these CV outcomes. Cardiorespiratory fitness, not WC or visceral fat, was found to be the most important predictor of central DBP and AP suggesting a possible protective effect of increased levels of fitness against visceral fat and WC.

It has been shown that individuals who gain following weight loss are more than likely to increase fat mass than restore fat-free tissue.^39–41^ As to why this occurs; there are some data to suggest a downregulation in resting energy expenditure following weight loss, leading to increased fat accumulation.^42^ The current study measured resting metabolism, using indirect calorimetry (data not presented), and found no significant association with resting metabolism and measures of WC. Therefore, other possible mechanisms need exploration as to the increased levels of fatness associated with WC.

It is well understood that abdominal obesity is associated with increased risk of cardiometabolic abnormalities.^43,44^ A theory has been suggested that the deleterious effects of WC could be a result of a redistribution of body fat to more of an android shape.^13,15^ Indeed, in the current study it was found that WC had greater correlation values with visceral fat than total body fat measured using iDXA. While animal models support the hypothesis that cycles of starving and refeeding cause a preferential storage of visceral fat,^45,46^ human models are inconclusive.^16,42,47^

Montani et al.^42^ proposed the theory of an overactive sympathoadrenal system, secondary to visceral adiposity,^48^ as a possible mechanism for the risks of weight regain on CV health. The current study did not measure indicators of the sympathetic nervous system; however, Masuo et al. showed that weight gain caused an increased activation of the sympathetic nervous system.^49^ Our results collaborate with what has been done illustrating a link between visceral fat and CV measures and further provide a framework showing the link between WC and CV dysfunction through the mediator of visceral fat.

Many studies have provided evidence that obese subjects with an increased cardiorespiratory fitness have lower all-cause mortality and lower risk of CV and metabolic diseases compared to leaner unfit individuals.^50,51^ Indeed, higher fitness levels have shown to correlate with decreased arterial stiffness.^52^ Aerobic exercise training has also been shown to reduce resting BP 5–7 mm Hg among those with HTN.^53^ This magnitude of reduction rivals decreases obtained from first line antihypertensive medications and lowers heart disease risk by 20–30%.^54^ In fact, the literature suggests that the greatest drop in resting BP occurs after 1–3 weeks, independent of weight loss, of aerobic exercise training with no further decrease thereafter.^55–57^ A 2014 meta-analysis^58^ found that aerobic exercise significantly reduced PWV by −0.63 m/sec. This value could lead to an 8% reduction in CV events and a 9% reduction in CV mortality. It has been suggested that the obesity paradox may be partly explained by the level of cardiorespiratory fitness, which is rarely measured in obesity research.^59^ Our results support this notion that fitness was a stronger predictor than age, WC, or visceral fat on outcomes of central DBP and AP.

There are many strengths to the current study. Our assessment of visceral fat, using iDXA, is superior to a simple waist circumference measurement that some prior studies utilized.^60^ The measurement of arterial stiffness is also a strength to the current study. It is well accepted that arterial stiffness precedes the development of HTN.^31,32^ In our study it was found that measures of WC were not associated with peripheral BP but was associated with central BP and measures of arterial stiffness. Prior research in this area has focused on peripheral BP assuming that it represents central hemodynamics and arterial stiffness when in fact they may have missed deleterious effects of WC on these measures. This alone may explain the divergent findings in the literature when it relates to WC and BP.^18^ Different cohorts assuredly differ on the exposure time of WC (how fast weight was lost and regained, and how long before measurement did the last cycle occur) and, therefore, may not present with peripheral HTN as of yet but are in the stages of progression. This could only be investigated if measures of central BP and arterial stiffness are taken.

Another strength of the study was the use of a mediation analysis to provide a pathway for the progression of WC to increased BP. We have shown that WC is strongly associated with visceral fat accumulation, and visceral fat accumulation is strongly associated with measures of arterial stiffness. It was also shown that after adjusting for visceral fat, the association between WC and arterial stiffness dissipates, suggesting that visceral fat supplied full mediation between this link. In addition, the objective assessment of CV fitness is another strength of the current study and is rarely taken into account and/or measured in the WC literature.

The current study is not without weaknesses. The assessment of WC was self-reported and collected retrospectively. We do believe that the methods to obtain weight history however are reliable as shown in a prior test-retest assessment.^37^ Also it must be noted that there is no universal definition among clinicians and researchers on how to define a single weight cycle and who constitutes a weight cycler. In addition, study subjects were limited to middle aged, obese, and relatively unfit women. Thus, these relationships may not be generalized to other populations. Finally, it must be noted that there are many factors that can negatively impact arterial stiffness such as life stress and dietary patterns that were not considered in the current study. It must be left to future research with larger sample sizes to explore the independent effects of WC and visceral fat when controlling for these other factors.

In conclusion, with ∼57% of woman dieting at any given time^4^ and recidivism rates of weight loss ranging from 80%^6^ to 95%,^7^ WC is extremely prevalent. The current study has shown that WC may increase visceral fat accumulation, which is associated with increased central SBP and measures of arterial stiffness. It also appears that increasing fitness may be somewhat protective against the damaging effects of WC on visceral fat accumulation, BP, and arterial stiffness. From a societal prospective, it may be easier to get people to increase fitness than lose body mass and this approach may still increase the health of these individuals regardless of body weight status.

## Abbreviations Used

AIx: augmentation index
AP: augmentation pressure
BMI: body mass index
BP: blood pressure
cfPWV: carotid-femoral pulse wave velocity
CI: confidence interval
CV: cardiovascular
CVD: cardiovascular disease
DBP: diastolic blood pressure
iDXA: dual-energy X-ray absorptiometry
HR: heart rate
HTN: hypertension
PA: physical activity
PWA: pulse wave analysis
PWV: pulse wave velocity
SBP: systolic blood pressure
WALI: Weight and Lifestyle Inventory
WC: weight cycling
WCI: weight cycling index
